# Presynaptic Paraneoplastic Disorders of the Neuromuscular Junction: An Update

**DOI:** 10.3390/brainsci11081035

**Published:** 2021-08-03

**Authors:** Maria Pia Giannoccaro, Patrizia Avoni, Rocco Liguori

**Affiliations:** 1 Dipartimento di Scienze Biomediche e Neuromotorie, Università di Bologna, 40138 Bologna, Italy; mariapia.giannoccar2@unibo.it (M.P.G.); patrizia.avoni@unibo.it (P.A.); 2 IRCCS Istituto delle Scienze Neurologiche, UOC Clinica Neurologia, Ospedale Bellaria, Via Altura 3, 40139 Bologna, Italy

**Keywords:** Lambert Eaton myasthenic syndrome, neuromuscular junction, presynaptic disorders, paraneoplastic syndrome, neuromyotonia, CASPR2, immune checkpoint inhibitors

## Abstract

The neuromuscular junction (NMJ) is the target of a variety of immune-mediated disorders, usually classified as presynaptic and postsynaptic, according to the site of the antigenic target and consequently of the neuromuscular transmission alteration. Although less common than the classical autoimmune postsynaptic myasthenia gravis, presynaptic disorders are important to recognize due to the frequent association with cancer. Lambert Eaton myasthenic syndrome is due to a presynaptic failure to release acetylcholine, caused by antibodies to the presynaptic voltage-gated calcium channels. Acquired neuromyotonia is a condition characterized by nerve hyperexcitability often due to the presence of antibodies against proteins associated with voltage-gated potassium channels. This review will focus on the recent developments in the autoimmune presynaptic disorders of the NMJ.

## 1. Introduction

Paraneoplastic disorders are a group of diseases that develop as a consequence of an immune response against cancer. According to the classical model, the tumor cells express antigens which are shared by neuronal tissue that stimulates a T cell response directed against not only the tumor but also the nervous system. Many of these responses are accompanied also by the production of antibodies against intracellular antigens, defined as onconeural antibodies [1].

The NMJ is a highly specialized synapsis that connects the nerve with the muscle and is the target of several autoimmune disorders. On reaching the muscle, motor nerves lose their myelin sheath and branch into nerve endings forming synaptic boutons that contact the muscle surface. The gap between the presynaptic nerve terminal and the muscle, postsynaptic, membrane is called synaptic cleft and is filled with the synaptic basal lamina, a specialized extracellular matrix. The postsynaptic part of the NMJ is a specialized portion of the sarcolemma characterized by the presence of junctional folds [2].

The autoimmune disorders involving the NMJ are classified according to the site of the antigenic target and the consequent neurotransmission failure as presynaptic and postsynaptic. This review will focus on the recent developments in immune-mediated presynaptic disorders, including LEMS and acquired NMT.

## 2. The Neuromuscular Junction

The NMJ is a chemical synapsis, designed to transform the motor action potential into muscle fiber contraction [2]. The arrival of the motor action potential opens VGCCs located in the motor nerve terminal with consequent Ca^2+^ influx and exocytosis of the ACh stored in synaptic vesicles at the active zone of the presynaptic terminal. ACh binds to nicotinic AChR on the postsynaptic membrane. The AChR are ligand-gated cation channels that open when bound by two ACh, causing the influx of positively charged ions, mainly sodium, with the generation of an EPP. Besides ACh release induced by nerve activity, a motor nerve terminal spontaneously releases, at irregular intervals, single ACh quanta through exocytosis of single synaptic vesicles. The single ACh quantum leads to a postsynaptic miniature EPP [3].

**Figure 1:**
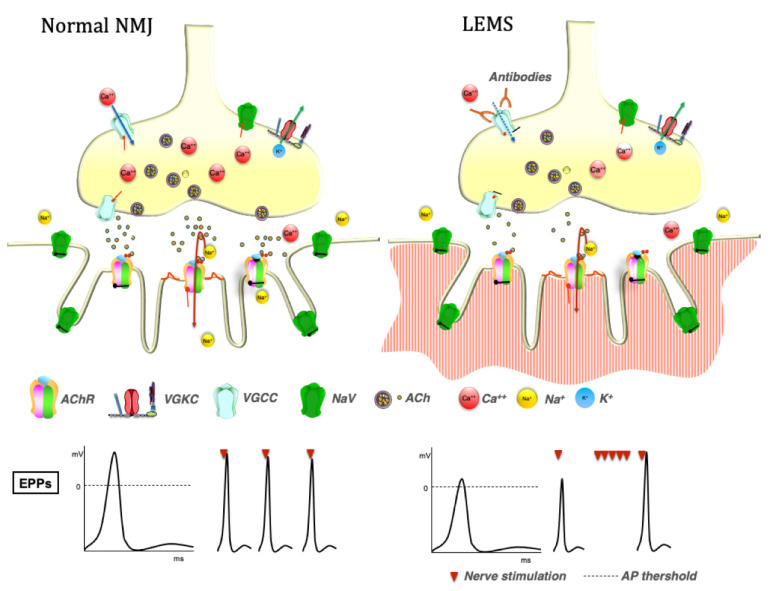
Pathophysiology of LEMS. In normal conditions, the depolarization of the presynaptic nerve terminal leads to calcium ions influx, acetylcholine (ACh) release, and binding to the ACh receptors (AChR) with a consequent influx of positively charged ions, mainly sodium, generation of an endplate potential (EPP) and muscle contraction. In LEMS, voltage gated calcium channel (VGCC) antibodies block calcium influx causing a reduction of the ACh released at the presynaptic terminal with consequent reduction of the EPP amplitude. High-frequency repetitive nerve stimulation however can increase the EPP amplitude through calcium accumulation in the presynaptic terminal and increased ACh release.

In health, the EPP is usually sufficient to reach the threshold for opening voltage-gated sodium channels generating an action potential that propagates along the muscle fiber leading to contraction (Figure 1). The extent to which the EPP exceeds the threshold for the generation of an action potential is called the “safety factor” of neuromuscular transmission, and it allows the NMJ to continue functioning under different conditions. The EPP is short-lived since both AChRs and VGCCs close spontaneously while ACh disperses by diffusion or is hydrolyzed by the AChE. The opening of VGKCs at the presynaptic membrane restores the membrane potential and limits the opening of VGCCs [3].

Both LEMS and NMT affect the safety factor, although with different mechanisms. In LEMS, VGCCs antibodies decrease the safety margin by decreasing ACh release. In NMT, antibodies against VGKC compromise the safety factor by causing delayed repolarization of the axon after each action potential and prolongation of the depolarization of the muscle fiber membrane [4].

## 3. Lambert-Eaton Myasthenic Syndrome (LEMS)

LEMS is a neuromuscular autoimmune disorder associated with the presence of antibodies against the presynaptic VGCCs. The association with cancer was recognized since the first description of the disease in 1956 [5] and LEMS has been classically classified into paraneoplastic (SCLC-LEMS) and non-paraneoplastic (NT-LEMS) [6]. Since some clinical features overlap those of MG, many cases are misdiagnosed or diagnosed later in the course of the disease. Although rare, the clinical recognition of LEMS is relevant as more than half of patients have an associated tumor, usually, SCLC and early disease recognition and cancer investigations are fundamental to ensure a good prognosis.

### 3.1. Immunology and Pathophysiology

LEMS is caused by antibodies targeting presynaptic VGCCs [7], multi-subunit channels that allow the transmembrane influx of calcium in response to an action potential. In vitro these antibodies can induce cross-linking and internalization of their target [8,9], ultimately interfering with the calcium flux required for ACh release [10] (Figure 1), and the passive transfer of IgG from affected individuals to mice reproduce some of the clinical features of the disease, suggesting a pathogenic role of the antibodies [8,11]. In rats, active immunization with peptides derived from the extracellular region of the α1 subunit of the VGCC showed some LEMS-like features [12].

Studies on HEK293 cells and cerebellar granular cells showed that these antibodies reduce the Cav2.1 (P-type) currents with a compensatory increase of the expression of other VGCCs [13]. In cultured hippocampal neurons, these antibodies were shown to impair vesicular exocytosis from synaptic boutons [14].

In the majority of LEMS patients, the antibodies target the alpha1A subunit of P/Q type VGCCs [7], the most abundant at the presynaptic nerve terminal. P/Q- type VGCCs are also expressed at autonomic synapses explaining the presence of autonomic symptoms [15] as well as on the cerebellum. More rarely, patients harbor antibodies against the N-type and L-type VGCCs [13,16,17], although their significance and clinical utility are still unclear, and they are usually associated with the presence of P/Q type antibodies [17,18,19]. About 10% of patients with LEMS have concomitant PCD [20,21], associated with the reduction of the P/Q type VGCCs in the molecular layer of the cerebellum [21] at neuropathology, suggestive of a pathogenic role of the P/Q type VGCC antibodies. Recently, GRP78 antibodies were detected in 83.3% of patients with PCD-LEMS compared to only 6.6% of patients with LEMS. Functional studies on human brain microvascular endothelial cells suggested that these autoantibodies could increase the blood-brain barrier permeability and facilitate cerebellar dysfunction in patients with PCD-LEMS [22].

Autoantibodies to other proteins have been described in patients with LEMS, such as synaptotagmin and muscarinic acetylcholine receptors m1, in both seropositive and seronegative LEMS [23,24,25,26].

About 10–15% of patients with LEMS are seronegative, although their clinical phenotype is very similar to that of seropositive cases [20]. A relevant difference is an association with SCLC, seen in about 70% of VGCC positive LEMS versus 12% of seronegative [20]. Passive transfer of seronegative LEMS sera to mice generate the same phenotype and electrophysiological changes observed in mice treated with antibody-positive sera [20], suggesting that seronegative LEMS might be due to VGCC antibodies but at a relatively lower titer, or antibodies directed against VGCCs’ epitopes not recognized by current assays [6].

In more than half of LEMS patients, the etiology is paraneoplastic. SCLC cells have been shown to express functional VGCCs on their surface membranes [27] and antibodies from LEMS patients can inhibit the function of these channels in a dose-dependent manner [28].

Differently from patients with cancer, the trigger of NT-LEMS is unknown. The HLA-DR3-B8 is present in around 69% of patients with NT-LEMS compared to 23% of the control group, and 12% of paraneoplastic LEMS [29,30]. This haplotype is linked to autoimmunity, and it has been also suggested that it could improve the immunosurveillance against SCLC [30]. The underlying genetic predisposition to autoimmunity in patients with NT-LEMS is also supported by the association with other autoimmune disorders such as thyroid disorders and type 1 diabetes [31,32] and with the presence of a variety of organ-specific antibodies [33].

### 3.2. Clinical Features and Diagnosis

LEMS is a rare disorder with a prevalence between 2.3 and 3.8 per million population [34,35,36], mainly affecting middle-aged adults, although rare cases have been reported also in children [37]. Initial reports described a male prevalence [38,39], but this is true only for the SCLC-LEMS, which affect mainly men with a mean age of 60 years [40]. The age of onset of NT-LEMS has a bimodal distribution, with a peak age of onset of around 35 years and a second peak at age 60 years [40]. NT-LEMS has a similar frequency in men and women although a female predominance has been observed in patients below the age of 45 years, and a male predominance in those diagnosed after the age of 60 years [31].

Clinically, LEMS is characterized by proximal muscle weakness, dysautonomia, and reduced or absent deep tendon reflexes, due to a reduced release of ACh from the motor nerve terminal. Characteristically, muscle strength, as well as deep tendon reflexes, can increase transiently after a forced muscle contraction, differentiating LEMS from MG. This feature, called post-exercise facilitation, can also be observed after high-frequency nerve stimulation [41]. Indeed, LEMS is characterized by reduced evoked endplate potential amplitude with normal miniature endplate potential amplitude, in accordance with a significant reduction in ACh release and normal postsynaptic sensitivity to ACh (Figure 1). However, HFRS can overcome the calcium channel blockade [41,42]. This effect is believed to be caused by calcium accumulation at the presynaptic terminals, leading to an increased probability of ACh release (Figure 1).

Muscle weakness involves mainly the lower extremities and usually progresses slowly over time. However, a subacute or acute presentation may occur, and progression tends to be faster in patients with cancer [43,44,45]. Weakness normally spreads proximally to distally, and caudally to cranially. Cranial muscle involvement has been reported in 30–65% of LEMS patients. Ptosis and diplopia are the most common manifestations [46,47,48], but oropharyngeal symptoms have also been observed [47,48]. Mild autonomic dysfunction is very common (80–96% of patients) and includes dry mouth, impotence, constipation, and orthostatic hypotension [38,45,49].

Diagnosis is based on clinical features, neurophysiology, and antibody testing. Due to the frequent mildness and the lack of specificity of initial symptoms, diagnostic delay, as well as misdiagnosis, are frequent. The median time to diagnosis is 4 months in SCLC-LEMS and 12–19 months in NT-LEMS [40,50] and a study showed that up to 58% of patients were initially misdiagnosed [40,45].

RNS is the neurophysiological study of choice for the diagnosis, and it should be performed in at least two muscles [51]. RNS usually shows reduced CMAP amplitudes at rest, which decrements further at low frequencies of repetitive stimulation (2 to 4 Hz). A decrease of CMAP amplitude (decrement) of at least 10% is considered abnormal [51]. The decremental pattern during low-frequency RNS might help distinguish between MG and LEMS. Indeed, in MG, a train of 8–10 stimuli produces a progressive decrement in CMAP response, which is usually maximal at the fourth or fifth stimulation while it increases, although remaining below the size of the initial response, with subsequent stimuli. On the contrary, in LEMS, a low-frequency RNS produces a progressively decremental response [52,53]. Moreover, immediately after brief exercise (or with HFRS at 25 Hz to 50 Hz), CMAP amplitude increases significantly (increment) in LEMS but not in MG, helping in the differential diagnosis between MG and LEMS. Post-exercise and high-frequency stimulation have similar sensitivity of 84–96% [54,55,56] but the first is preferable as it is less painful, although high-frequency stimulation could have a slightly higher diagnostic yield [54]. An increment higher than 100% is considered 100% specific for LEMS, but a cut-off of 60% has been proposed to improve sensitivity to 97%, while maintaining a 99% specificity [54].

VGCCs antibodies can be detected by radioimmunoprecipitation. P/Q-type and N-type VGCC are extracted from human or mammalian cerebellum that is labeled with 1251-ω-Conotoxin MVIIC or GVIA derived from the Conus genus of piscivorous snails [10,16]. P/Q type VGCC antibodies have been reported to be very sensitive and specific for the diagnosis of LEMS [16,17], although low titers could be found also in controls, in patients with various types of cancers as well as in patients with amyotrophic lateral sclerosis [16,57]. However, a recent study observed the presence of these antibodies in a high proportion of patients without a neurological autoimmune or inflammatory etiology, with no difference in antibody titers between the autoimmune and the not-autoimmune groups [58], confirming that the P/Q type VGCC antibodies are not diagnostic in the absence of clinical and electrophysiologic features of LEMS. No correlation has been found between antibody titers and clinical features or outcome, although antibody levels might decrease after immunosuppressive treatment [59,60].

### 3.3. Cancer Association

About 60% of LEMS are paraneoplastic, usually associated with SCLC [38] expressing P/Q type VGCCs [61]. Other malignancies observed in patients with LEMS include lymphoproliferative disorders [37,60,62,63,64], prostate carcinoma [44], Merkel cell carcinoma [65], different thymic cancers [66,67], neuroblastoma [37] and atypical carcinoid [68]. Although the co-occurrence could be casual, the association with some of these tumors, including lymphoproliferative disorders, prostate cancer, and MCC, suggests causality. LEMS has been observed in patients with lymphomas and leukemias [37,60,62,63,64]. In these cases, LEMS symptoms could precede or follow the cancer diagnosis. In patients with prostate cancer, the tumor had neuroendocrine and small cell characteristics, and LEMS symptoms correlated with cancer activity [44]. MCC is an aggressive neuroendocrine tumor classified as an extrapulmonary SCLC, from which is almost histologically indistinguishable [69]. Eight cases of MCC-associated LEMS have been reported so far [65,69,70]. Most cases improved with oncological treatment and immunotherapy.

VGCC-antibody positivity does not distinguish patients with paraneoplastic LEMS however, the presence of other antibodies can help to predict the presence of cancer. In particular, SOX antibodies have been reported in 64% of patients with SCLC [71]. Moreover, N-type VGCC and GABAB receptor antibodies are associated with a higher risk of SCLC-LEMS [72]. A clinical score, the DELTA-P score, based on demographic and clinical features, can help to identify patients at high risk for SCLC who require more extensive screening. A score between 3 and 6 is associated with a probability of SCLC of 83.9–100% [40]. The presence of additional neuronal antibodies could warrant a more intensive SCLC screening in patients with DELTA-P scores of 1 or 2, which are associated with a low risk of SLCL [72]. All patients with a novel diagnosis of LEMS and no history of cancer should undergo an initial oncologic screening with a CT scan of the chest, abdomen, and pelvis. If negative, CT should be followed by 18F-PET. In case of negativity, surveillance should be continued at 3 or 6-month intervals for at least 2 years, based on the DELTA-P score and additional risk factors [40,72].

Rarely, LEMS has been reported as an adverse event of cancer treatment in patients receiving immune checkpoint inhibitors therapy [73,74,75,76,77]. These are a group of monoclonal antibodies directed against immune cell receptors expressed on T cells or their ligand, which aim at boosting the anti-tumor activity of cytotoxic T cells by interfering with inhibiting signals that reduce their activity [78]. The main targets are CTLA-4 and the PD-1 or its ligand, PD-L1. Immune-related adverse events (irAE) have been observed with every class of ICI and are related to the promotion of cellular- and antibody-mediated autoimmunity, although the exact underlying pathophysiological mechanisms remains only partially understood and several factors may play a role [79]. LEMS has been reported after treatment with different ICI (nivolumab, pembrolizumab, and ipilimumab) for different cancers, including SCLC [73,75,77], squamous cell lung carcinoma [74], and neuroendocrine tumor [76]. Response to immunotherapy, 3,4-diaminopyridine (3,4-DAP), and ICI withdrawal were variable. Diagnosis and treatments for ICI-associated LEMS may be challenging, particularly in patients with SCLC. However, in these cases, differentiating between irAE and paraneoplastic etiology is relevant as treatment can differ and affect the cancer outcome. Indeed, high-dose steroids are considered the first-line treatment for most neuromuscular irAEs [80,81]. Although contrasting results have been reported so far [82,83,84], steroids might negatively affect cancer outcomes, at least in patients with SCLC [83]. Moreover, patients with irAE should suspend ICI therapy in severe cases and consider subsequent re-challenge, whereas patients with SCLC-LEMS can continue the treatment. There are not, at present, biomarkers that help to identify patients with irAE vs paraneoplastic LEMS. The time of onset could be relevant in helping the differential diagnosis, since in SCLC-LEMS symptoms usually precede the cancer diagnosis [85], whereas the median time of irAE onset is 19 weeks from ICI treatment initiation [86]. With the expanding approved indication of ICI treatment, more cases of irAE-LEMS will likely be reported in the future, helping the identification of clues to their correct identification and management.

### 3.4. Treatment and Prognosis

LEMS treatment is based on symptomatic therapy and immunosuppression. In patients with cancer, oncologic treatment is of the highest priority. Cancer treatment usually leads to a clinical improvement, although, in cases with residual symptoms, additional treatment might bring further benefit. The treatment choice is based on the clinical severity of the disease. Recently, a new measure, the Triple Timed-Up-and-Go test, has been established and validated for assessing clinical function and the outcome in patients with LEMS that could be used to guide treatment [87,88].

**Figure 2:**
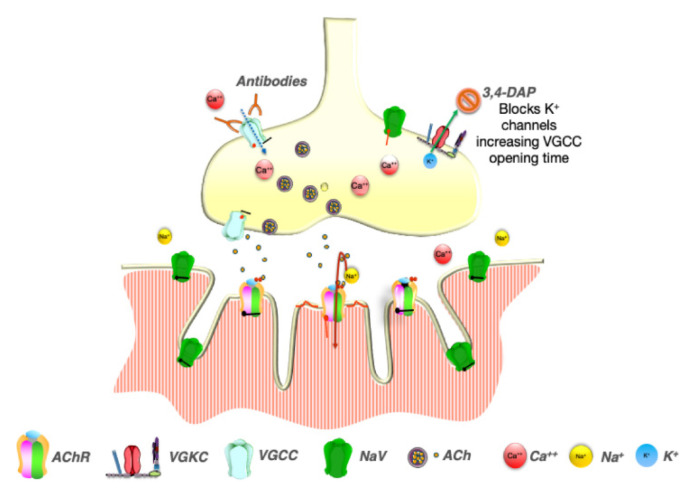
Effect of 3,4-diamniopyride treatment in LEMS. 3,4-diaminopyridine (3,4-DAP) blocks the efflux of potassium (K+) prolonging the presynaptic depolarization, which in turn prolongs the VGCCs opening time, allowing the influx of an increased amount of calcium ions with consequently increased ACh release.

Independently from the tumor presence, patients with moderate or severe weakness interfering with function, should be started on symptomatic treatment. The treatment of choice is amifampridine (3,4-DAP), which acts by blocking the potassium channels Kv3.3, prolonging the presynaptic nerve terminal membrane depolarization, therefore enhancing the calcium entry, and improving the ACh release (Figure 2).

More recently, a further mechanism of action, a direct agonistic effect of 3,4-DAP on Cav1 type channels, has been reported, although its relevance at therapeutic concentrations is debated [89,90,91]. Several randomized controlled trials demonstrated the superiority of 3,4-DAP compared to placebo in improving motor and autonomic symptoms [92,93,94,95,96,97,98,99], and 3,4-DAP use in LEMS has been approved in 2009 in Europe and 2018 in the US. The starting dose is 15 to 30 mg daily in adults and 7.5 to 15 mg in children, divided into 3-4 doses. The maximum approved daily dose is 80 mg in adults and 50 mg in children. The most common side effects are oral (40%) and digital (34%) paresthesia, followed by headache, nausea, and diarrhea (9.4%) [97]. High doses of 3,4-DAP have been associated with seizures and Q-T interval prolongation [92,100] and its use is contraindicated in patients with epilepsy [100]. In patients with residual or refractory weakness, immunosuppressive therapy should be introduced. Oral prednisone, alone or in combination with AZA, has been shown to be effective in a retrospective study [101]. However, IVIG is suggested as first-line treatment [102] in patients with LEMS, based on case reports [103,104] and a placebo-controlled crossover trial showing significant improvement in limb strength after treatment [59]. PLEX has been reported as LEMS treatment in case reports/series but there are not clinical trials [101,105,106]. Rituximab is suggested in patients refractory to other treatments [107,108].

Survival is shorter in patients with SCLC-LEMS and depends on the disease stage at presentation. On the other hand, LEMS is associated with longer survival in patients with SCLC [109,110,111,112] with a median of 17.3 months in these patients versus 10 in those with SCLC without neurologic symptoms [111]. Given the rarity of LEMS in association with tumors other than SCLC, there are no data on the prognosis of this group of paraneoplastic patients. In patients with NT-LEMS, life expectancy appears comparable to that of the general population [112]. Overall, functional impairment tends to be more severe in patients with paraneoplastic LEMS, although a recent study showed that the health-related quality of life does not differ significantly between patients with SCLC- and NT-LEMS [112]. A follow-up study on 47 patients with LEMS showed that 88% had an improvement of muscle strength whereas a sustained clinical remission was achieved in 43%. About a quarter of patients needed a wheelchair, at least outside the house, and more rarely, at all times [60]. In a more recent study, 62% were independent for daily living activities at diagnosis, and 85% at the 1-year follow-up. During the disease course, 73% of patients used any device to assist mobility; 52% used a wheelchair, but only 6% were fully wheelchair dependent [112].

## 4. Neuromyotonia (NMT)

Peripheral nerve hyperexcitability includes a spectrum of clinical manifestations ranging from cramp fasciculation syndrome to NMT, characterized by involuntary muscle activity caused by spontaneous discharges originating from the motor nerve fibers [113]. Neuromyotonia or Isaacs’ syndrome was firstly reported in 1961 by Dr Hyam Isaacs, who described a ‘syndrome of continuous muscle-fiber activity’ characterized by progressive muscle stiffness, with widespread fasciculation leading to weakness [114]. The term neuromyotonia was coined in 1965 by Mertens and Zschocke to underline the myotonia-like spasm and the origin from the peripheral nerve [115]. NMT can be hereditary or acquired. Here we will focus on the autoimmune form.

### 4.1. Immunology and Pathophysiology

**Figure 3:**
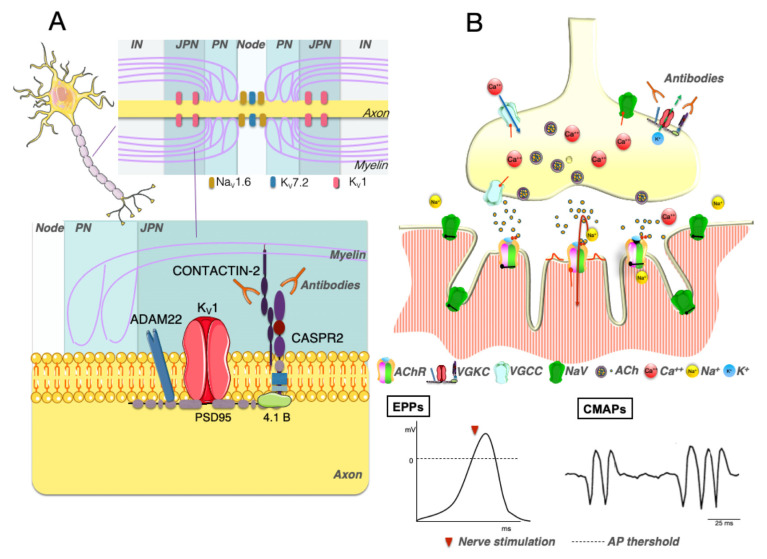
Pathophysiology of neuromyotonia. (**A**) Schematic overview of the VGKC complex in the juxtaparanodal region of myelinated axons (modified from [144]). CASPR2, together with contactin-2 and other proteins, is fundamental for clustering Kv1 channels. CASPR2 antibodies could interfere with CASPR2-contactin-2 interaction leading to Kv expression down-regulation with consequent impairment of action potential repolarisation and excessive or prolonged release of acetylcholine. (**B**) This leads to prolonged EPP and repetitive nerve firing. The EMG shows myokymic discharges (doublets, triplets, and multiplets).

Indirect evidence for an autoimmune etiology of NMT came from the demonstration that passive transfer of sera from affected patients into mice produced a raised threshold to d-tubocurarine in the diaphragm muscle and that PLEX significantly reduced the number of neuromyotonic discharges in patients [116,117]. In 1995, Shillito P et al. [118] developed a radioimmunoprecipitation assay using Kv1 type VGKCs extracted by the mammalian brain and labeled with iodinated alpha-dendrotoxin (125I-αDTX), a neurotoxin derived from the venom of the Dendroaspis angusticeps (Green mamba snake) which binds specifically to the Kv1.1, 1.2 and 1.6 subtypes of the Shaker channel family [119]. The authors found that three of six patients studied precipitated 125I-αDTX counts that were significantly higher than controls levels [118], indicating the presence of antibodies directed against the VGKC or related proteins. These channels functions in terminating presynaptic action potentials, therefore a reduction of their function induced by the antibodies, as shown by in vitro studies [120,121], would increase Ca2+ influx and impair action potential repolarization, leading to excessive release of AChR and NMT (Figure 3).

VGKCs antibodies were later found in a 76-year-old man with MoS [122] and shortly after in a few patients with LE [123,124,125]. Subsequently, it was discovered that the VGKCs-antibodies are not directed against the potassium channel but to associated proteins, including LGI1, CASPR2, and contactin-2 [126]. CASPR2 antibodies do not cause internalization of their target [127,128] but the passive transfer of CASPR2 immunoglobulin to mice produced nerve hyperexcitability and mechanical allodynia with loss of surface CASPR2 and VGKC Kv1 in the dorsal root ganglia [129]. LGI1 animal models did not explore the peripheral effects of the antibodies, however, both in vitro and in vivo studies showed that LGI1 antibodies inhibited the interaction between LGI1 and ADAM22/23 and reversibly reduced synaptic AMPA receptor clusters in hippocampal neurons [130,131].

The frequency of VGKC-complex antibodies in patients with NMT and related disorders ranged from 2 to 40% of cases [132,133,134,135]. In a more recent study including only NMT cases, 45% (17/38) of patients had specific antibodies (13% to CASPR2, 13% to contactin 2 (in one case associated with CASPR2), 5% to LGI1, and 16% to both CASPR2 and LGI1 [136]. Patients with LGI1-CASPR2 co-reactivity had a higher disability as detected by modified Rankin Scale, pain, dysautonomia, and sleep disorders, and thymoma [136], associations confirmed in another study [137].

About 30% of patients with CASPR2 or LGI1 have other autoimmune disorders, including MG and thyroid disorders [138], in accordance with the presence of strong HLA associations in patients developing LGI1 and/or CASPR2 antibodies [138,139]. Further observations suggested that the HLA haplotype could be relevant in determining the associated clinical phenotype and the underlying pathogenetic mechanisms in CASPR2 autoimmunity. Indeed, the HLA-DRB1*11:01, initially observed in 48% of patients with CASPR2 antibodies [138], was recently shown to be present in 93% of LE genotyped cases, but at the same rates as controls in patients with a prevalent PNH phenotype [137].

Rarely, other antibodies have been observed in patients with NMT, including collapsing response-mediator protein-5 [140], glutamic acid decarboxylase [141], muscle-specific tyrosine kinase [142], and immunoglobulin-like cell adhesion molecule 5 [143] antibodies, although their role is unclear.

Overall, about half of the patients with NMT are seronegative [133,136], suggesting the presence of other possible antibodies in these cases yet to be discovered.

### 4.2. Clinical Features and Diagnosis

NMT is a rare disorder characterized by spontaneous and continuous muscle activity at rest, which manifests clinically as visible muscle twitching (90%), cramps (70%), myopathy, stiffness, hypertrophy, and impaired muscle relaxation [113]. The twitches consist of myokymia, the irregular undulation of muscle fibers, resembling worms moving beneath the skin. Pseudomyotonia manifests as delayed relaxation after maximal voluntary contraction, typically elicited during a tight handgrip or eye closure [145]. Fasciculations can also be present. NMT usually starts insidiously, over months, mainly in the extremities, although the facial and truncal muscles can be involved as well [113]. The continuous muscle fiber activity is considered the cause of the hypertrophic appearance of the muscles in some patients. Muscle stiffness can be relevant, causing walking impairment, hyperlordosis, and respiratory difficulty [113,146]. Other frequently associated features include sensory symptoms such as paresthesia and pain and autonomic disturbances including hyperhidrosis, tachycardia, and diarrhea [136]. Sleep disorders, particularly insomnia, are not infrequent [136]. In its extreme form, NMT associates with signs of the central nervous system in the MoS characterized by the association of neuromyotonia with encephalopathy, sleep disorders (mostly insomnia), and dysautonomia [122].

Although epidemiological data are scarce, acquired NMT, is mainly observed in adult male patients, in the mid-40s [133,147,148]. However, few pediatric cases, associated with LGI1/CASPR2 antibodies have been reported [149,150]. Differently from adult cases, a tumor association has not been found in children.

The diagnosis of NMT is based on electrophysiological studies showing fibrillation and fasciculation potentials, myokymia, and neuromyotonic discharges, characterized by repetitive bursts of potential with short interpotential intervals (Figure 3b). Repetitive afterdischarges can be seen after the CMAP of nerve conduction studies [113]. The persistence of the abnormal nerve activity after peripheral nerve block, along with its disappearance after curare, suggests it originates from the distal motor axon or nerve terminal [114], although a proximal origin of discharges has been recorded as well [151].

Acquired neuromyotonia can occur as an idiopathic or paraneoplastic autoimmune disorder. About 25% of patients with acquired NMT have an associated tumor [133,136], supporting the need for an accurate malignancy screening in these cases. CASPR2 antibodies, in particular, are associated with thymoma in 20% of cases [152]. Rarer is the association with SLCL and lymphoma [133]. Recently, antibodies to the Netrin-1 receptors deleted in colon cancer and uncoordinated-5A, have been associated with the presence of thymoma in patients with CASPR2 antibodies, PNH, and MG [137,153,154]. The expression of Netrin-1 receptors and CASPR2 was confirmed in thymoma samples, similar to what had previously been observed for other antigens involved in paraneoplastic autoimmunity (i.e., titin) [155].

### 4.3. Treatment and Prognosis

In patients with an underlying tumor, oncologic treatment might induce an improvement of symptoms [117]. There are no approved symptomatic treatments for NMT. Frequently employed drugs are antiepileptics, particularly sodium-channel antagonists such as carbamazepine, sodium valproate, and phenytoin [136]. Other drugs, employed with variable success, include acetazolamide, gabapentin, lamotrigine, clonazepam, and mexiletine [145,156,157,158]. PLEX is associated with clinical and electrophysiological improvement as well as reduction of antibody titers [117]. IVIGs, steroids, AZA, methotrexate, and more recently, rituximab have been proposed as alternatives treatments [159,160].

Hypersensitivity to muscle relaxants has been suggested in patients with NMT, requiring caution during general anesthesia, and possibly lower doses of muscle relaxants, particularly when using non-depolarizing drugs [161].

## 5. Conclusions

LEMS and NMT are rare disorders, and the clinician’s suspicion is pivotal to pursue the proper diagnostic test and achieve a correct diagnosis. Both disorders remain under-recognized and diagnostic delays are frequent. Once the diagnosis has been established, oncological screening and appropriate treatment are fundamental for patients’ prognosis and quality of life. Although vast improvements in treatment and care have been made, further studies are needed to standardize immune therapy and improve the long-term outcome.

## Abbreviations

3:4-DAP, 3,4-diaminopyridine; 18F-PET, 18F-fluorodeoxyglucose-positron emission tomography; ACh, Achetilcholine; AChE, ACh esterase; AchR, ACh receptors; ADAM22/23, ADAM Metallopeptidase Domain 22/23; AMPA, α-amino-3-hydroxy-5-methyl-4-isoxazolepropionic acid; AZA, azathioprine; CASPR2, contactin-associated protein 2; CMAP, compound muscle action potential; CT, computed tomography; CTLA-4, cytotoxic T-lymphocyte antigen 4; DELTA-P, Dutch-English LEMS Tumor Association Prediction score; EPP, endplate potential; GABA(B), gamma-aminobutyric acid; GRP78, 78-kDa glucose-regulated protein; HEK293, Human embryonic kidney 293 cells; HFRS, high frequency repetitive stimulation; HLA, human leucocyte antigen; ICI, immune checkpoint inhibitors; irAE, immune-related adverse events; IVIG, intravenous immunoglobulin; LE, limbic encephalitis; LEMS, Lambert Eaton myasthenic syndrome; LGI1, leucine-rich glioma inactivated protein 1; MCC, Merkel cell carcinoma; MG, myasthenia gravis; MoS, Morvan’s syndrome; NMJ, neuromuscular junction; NMT, neuromyotonia; NT-LEMS, non-tumour LEMS; PCD, paraneoplastic cerebellar degeneration; PLEX, plasma exchange; PNH, peripheral nerve hyperexcitability; PD-1, programmed cell death-1; PD-1L, programmed cell death-1 ligand; RNS, repetitive nerve stimulation; SCLC, small-cell lung carcinoma; SCLC-LEMS, paraneoplastic LEMS; SOX, SRY-related high mobility group [HMG] box; VGCC, voltage gated calcium channels; VGKCs, voltage-gated potassium channels.
